# Glass forming ability and critical exponents in Hf-Modified Fe-Zr-B-Cu amorphous alloys for near room temperature magnetocaloric application

**DOI:** 10.1038/s41598-025-08371-x

**Published:** 2025-07-07

**Authors:** Anjana Vinod, D. Arvindha Babu, N. V. Rama Rao, M. M. Raja, K. Guruvidyathri, S. Srinath, W. Madhuri

**Affiliations:** 1 School of Advanced Sciences, Vellore Institute of Technology, Vellore, 632014 Tamil Nadu India; 2 Defence Metallurgical Research Laboratory, Hyderabad, 500058 Telangana India; 3 University of Hyderabad, Hyderabad, 500 046 India; 4 Ceramic Composites Laboratory, Centre for Functional Materials, Vellore Institute of Technology, Vellore, Tamil Nadu India

**Keywords:** Fe-Zr-B-Cu, Hf substitution, Magnetocaloric effect, Critical behaviour, Mean-field model, Widom scaling relation, Applied physics, Materials science

## Abstract

Rare earth free magnetocaloric materials have emerged as a potential candidate for application in magnetic refrigeration presented in this work. A systematic investigation of Hafnium (Hf) substitution effects on the structural, thermal, magnetic, and magnetocaloric properties of rare earth free Fe-Zr-B-Cu alloys, synthesized via arc melting and melt spinning is reported in the present work. Comprehensive characterization using X-ray diffraction, differential scanning calorimetry, and vibrating sample magnetometry reveals enhanced magnetocaloric performance, with magnetic entropy change $\:(\:-{\varDelta\:S}_{M}^{max})$ ≈ 1.856 J/kg K, 1.767 J/kg K, full-width at half-maximum ($\:{{\Delta\:}T}_{FWHM}$) of 12.59 K, 31.98 K, and relative cooling power $\:\left(RCP\right)$ ≈ 23.36 J/kg, 56.50 J/kg under 2.5 T for Fe_88_Zr_3_Hf_4_B_4_Cu_1_ ,Fe_88_Zr_1_Hf_6_B_4_Cu_1_ alloys respectively. The analysis of critical exponents substantiates the occurrence of a second-order phase transition from paramagnetic to ferromagnetic at $\:{T}_{C}$ = 293 K for Fe_88_Zr_3_Hf_4_B_4_Cu_1_ alloys and $\:{T}_{C}$ = 303 for Fe_88_Zr_1_Hf_6_B_4_Cu_1_ alloys, in accordance with the mean-field model and the Widom scaling relation. These findings demonstrate the potential of Hf substitution in optimizing magnetocaloric properties and elucidating critical behavior, paving the way for advanced magnetic refrigeration materials.

## Introduction

Refrigeration is an important constituent of modern society, the process of refrigeration underpins global food security, public health, and economic development^1^. About 30% of the world’s food production needs refrigeration to avoid deterioration, nutritional losses, and risk of unsafe foods^2^. Refrigeration is important for healthcare beyond food security, as precise temperature control is necessary to keep pharmaceuticals, vaccines, and medical supplies cold^3^. In fact, conventional refrigeration systems are battling against some serious challenges themselves, as they utilize hydrofluorocarbons (HFCs), and chlorofluorocarbons (CFCs), which directly contribute to climate change, create holes in the ozone layer and cause more environmental damage^4^. These systems are energy-intensive, accounting for 17% of world consumption, and are subject to limitations in efficiency, dependability, and scalability^5^. The Kigali Amendment aims for an 80% decrease in HFC emissions by 2050, necessitating the development of innovative technologies to supplant conventional systems^6^. The electrically powered magnetocaloric effect is a cutting-edge refrigeration technology that provides a sustainable, efficient, and eco-friendly solution with a substantially reduced carbon footprint^7^. Magnetic refrigeration systems are capable of being more efficient (by up to 30%), quieter, smaller, and scalable, which enables them to be tailored to a variety of applications^8^. Magnetic refrigeration systems can be more efficient (by as much as 30%), quieter, smaller, and scalable, which allows for multiple uses^9^. Magnetic refrigeration has the ability to cut carbon emissions and energy costs by up to 50% through reduced energy consumption. So, magnetic refrigeration can be a game-changer for cooling technologies in the future because it presents a sustainable alternative to cater to the increasing global refrigeration needs as research progresses^10–12^.

Magnetic refrigeration utilizes magnetocaloric effect (MCE) which is a reversible magnetic field-induced temperature change in the materials of the solid state. Such a phenomenon takes place in magnetism when we have magnetic material, phase transition in it, and thus a change of its magnetic entropy^10^. When a magnetic field is applied, the magnetic moments of the material align resulting in a decrease of magnetic entropy and an increase in lattice entropy, leading to cooling. Secondly, an increase in magnetic entropy results in a decrease in the magnetic field, which attracts heat from the environment^13,14^. The efficient transfer of heat and chilling is facilitated by this repetitive process. Magnetisation, isotherm demagnetisation, demagnetisation, and isotherm magnetisation comprise the magnetic refrigeration cycle’s four stages. Magnetization cools the magnetic material under a rising external magnetic field^15–17^. Then, isothermal demagnetization occurs with the removal of the magnetic field, and heat transfers to the environment. As the material reverts to its original state it takes in heat a process known as demagnetization. The cycle is completed by restoring the magnetic field through an isothermal magnetization. Materials with large MCE, high thermal conductivity, and low hysteresis loss are critical factors to achieve optimal magnetic refrigeration performance. Studies center on locating and utilizing these materials, bringing magnetic refrigeration a step closer to an efficient, sustainable cooling solution^18^.

To attain efficient cooling, magnetic refrigeration technology requires materials with an optimal magnetocaloric effect (MCE)^19^. Traditional magnetocaloric materials, particularly those derived from rare earth elements^20–24^face limitations due to their elevated cost, toxicity, and restricted availability^25^. To promote sustainable, economical, and eco-friendly technologies, it is essential to create rare earth-free magnetocaloric materials. Conventional magnetocaloric materials heavily rely on rare earth elements, which are not only costly and prone to price volatility but also raise considerable environmental and social issues due to their extraction and processing^26,27^. Alternatively, magnetocaloric materials lacking rare earth elements offer a more environmentally sustainable option, enabling the development of novel applications in energy harvesting, refrigeration, and improved magnetic electronics. The reduced dependence on rare earth elements enhances the creation of more efficient and compact devices, alleviates risks linked to price volatility, and bolsters supply chain security^28^. The analysis highlights the importance of rare earth-free magnetocaloric materials in their potential to revolutionize various industries, including aerospace, energy, and healthcare, by providing a more sustainable, economical, and efficient option for magnetocaloric applications. The utilisation of iron-based alloys, specifically the Iron-Zirconium-Boron-Copper (Fe-Zr-B-Cu) alloy, has arisen as a potentially advantageous alternative^29,30^. These alloys offer a distinctive combination of high thermal stability, moderate toxicity, and a large MCE. Increases in magnetic moment, decreases in magnetic anisotropy, and improvements in mechanical properties are all brought about by the addition of Zr, B, and Cu to Fe^31^.

Fe-Zr-B-Cu alloy exhibits a tunable Curie transition temperature ($\:{T}_{C}$) near room temperature, crucial for magnetic refrigeration applications^29^. Its large MCE, high electrical resistivity, and low hysteresis loss ensure efficient heat transfer and minimal energy dissipation. Furthermore, Fe-Zr-B-Cu alloys corrosion resistance, mechanical strength, and scalability make it suitable for industrial applications. Research on Fe-Zr-B-Cu aims to optimize its composition, microstructure, and processing conditions to enhance MCE, $\:{T}_{C}$, and overall performance^32^. The partial substitution of Hafnium (Hf) for Zirconium (Zr) in Fe-Zr-B-Cu is expected to yield several benefits, including improved thermal stability, enhanced magnetic moment, and reduced magnetic anisotropy^33^. Specifically, Hf substitution can increase the alloy’s $\:{T}_{C}$, shifting it closer to room temperature, and amplifying the MCE, leading to enhanced cooling efficiency. Moreover, the addition of Hf can adjust the alloy microstructure, lessen defects, and enhance mechanical properties^34,35^. This takes a look at a specialty of investigating the impact of Hf substitution on the structural, magnetic, and magnetocaloric properties of Fe-Zr-B-Cu, to optimize its composition for high-performance magnetic refrigeration applications.

Magnetocaloric materials are crucial for magnetic refrigeration technology and exhibit intricate phase transitions that are in close proximity to their Curie transition temperatures ($\:{T}_{C}$)^36^. The magnetic ordering transition in the FeZrHfBCu alloy matrix is complicated and closely associated with critical magnetic behavior. Deciphering this complexity necessitates a profound comprehension of the fundamental interaction mechanisms.

Examining key exponents presents a viable method for attaining this comprehension. By analysing these exponents, researchers can investigate the interaction mechanisms occurring at the Curie transition temperature, elucidating the magnetic phase shift and its related dynamics^37^. Examination of critical exponents reveals the material’s intrinsic properties, including lattice dynamics, exchange interactions, and magnetic moment^38^The identification of optimal compositions, prediction of material behavior under various conditions, and enhancement of the magnetocaloric effect (MCE) are all facilitated by the investigation of critical exponents in magnetocaloric materials. Critical exponents also enable the comparison of theoretical models and experimental results, thereby facilitating the development of more precise theories. Additionally, the design of materials with customized properties, including thermal stability, MCE, and $\:{T}_{C}$, is influenced by a comprehensive comprehension of critical exponents.

This research is dedicated to the experimental determination and theoretical analysis of critical exponents in prominent magnetocaloric materials. The objective is to illuminate the fundamental physics that controls their behavior and to inform strategies for enhanced performance. This study systematically investigates the impact of Hf substitution on the FeZrBCu alloy matrix at the Zr site, focussing on structural, thermal, magnetic, and magnetocaloric properties. Additionally, a comprehensive analysis of the critical exponent is provided to evaluate the suitability of these materials for magnetic refrigeration applications at room temperature.

## Experimental methods

Fe-Zr-Hf-B-Cu (Fe_88_Zr_3_Hf_4_B_4_Cu_1_ and Fe_88_Zr_1_Hf_6_B_4_Cu_1_, Hereafter Hf4 and Hf6) alloys have been synthesized using arc melting beneath a high-purity argon environment. The melting process was repeated four times to guarantee the homogeneity of the alloys. Rapid solidification was performed using a single-roller melt-spinning device running in a high-purity Argon environment. The melt spinning process was performed with optimized parameters: a crucible inner diameter of 0.7 mm (nozzle) and 20 mm (melt pool), Argon pressure ranging from 10^−6^ bar, a wheel diameter of 30 cm, and a wheel speed of 50 m/s. A sample weight of 20 g and an input power of 10 kW were used in the process. These carefully controlled conditions resulted in rapid solidification and reduced contamination. The combination of a fast-rotating roller and precisely controlled melt-spinning conditions enabled ribbons to be produced by rapid solidification. Strict parameter management ensured the production of high-quality amorphous ribbons, characterized by uniformity and composition symmetry. The crystal structure of the samples was investigated by room temperature X-ray diffraction (XRD). The measurements were carried out using a Pan-analytical X-ray powder diffractometer. The measurements were conducted using Cu-Kα radiation at a wavelength of 1.5406 Å. Differential scanning calorimetry (DSC) measurements were carried out on TA instruments htdsc to determine the Glass Forming Ability (GFA) of the samples. The experiments were carried out in an argon atmosphere at a heating rate of 20 K/min. Thermomagnetic curves (M versus T) and isothermal magnetization curves (M versus H) were measured using a vibrating sample magnetometer (VSM) on Lake Shore cryotronics, USAmodel 7400 series VSM instrument. The measurements were performed up to an applied magnetic field of µ_₀_H = 2.5 T.

## Results and discussion

### Structural analysis

**Fig. 1:**
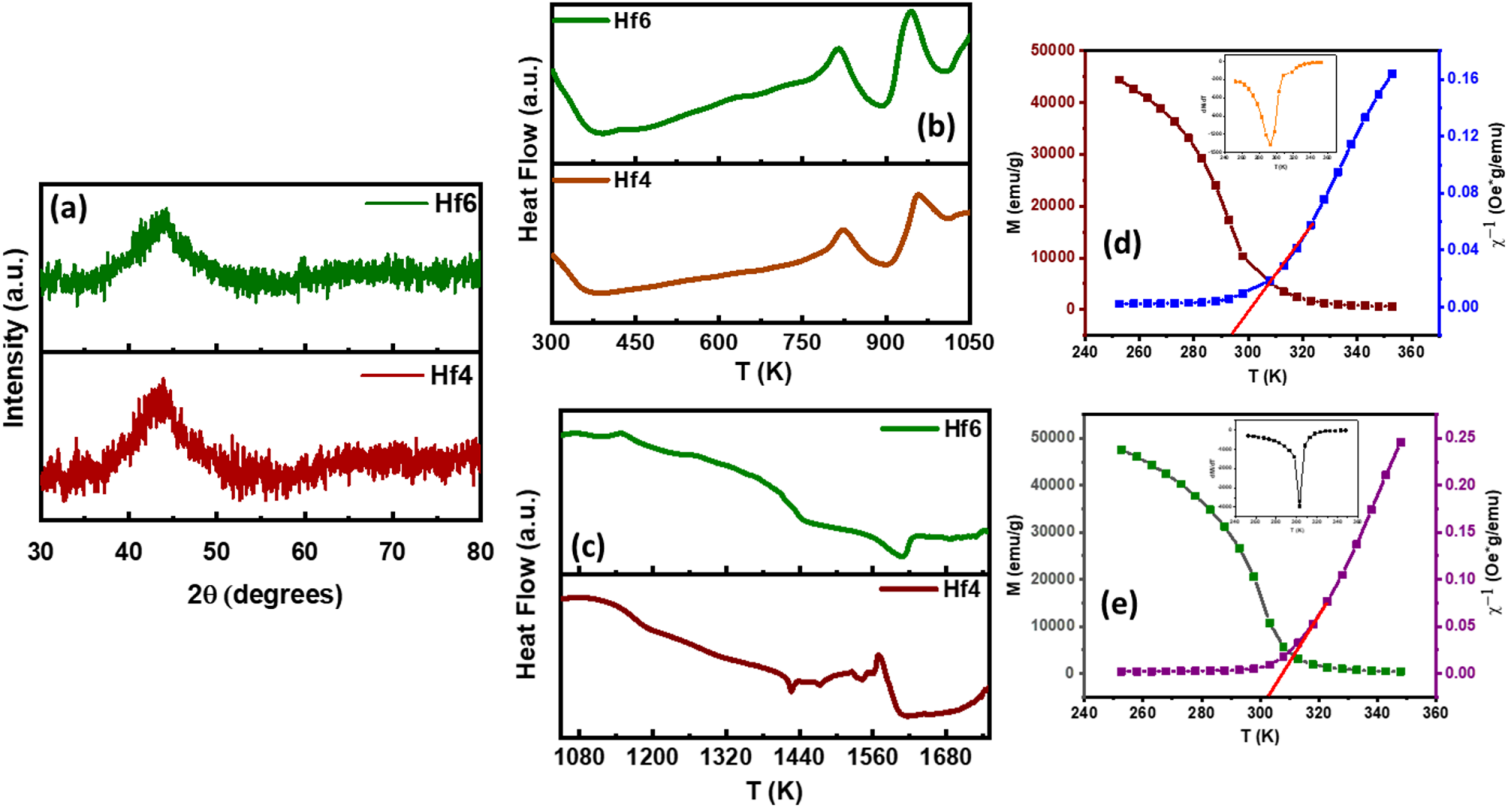
**(a)** XRD data of Hf4 and Hf6 ribbons **(b)-(c)** DSC data of Hf4 and Hf6 ribbons **(d)-(e)** M-T data and magnetic susceptibility, inset images depicted the derivative of the M-T data of Hf4 and Hf6 ribbons.

The X-ray diffraction (XRD) patterns of Hf4 and Hf6 alloys are graphically represented in Fig. 1(a). The XRD profile of Hf4 and Hf6 alloys exhibit pure amorphous structure. Melt-spun ribbons exhibit a consistent cross-sectional dimension of 2 mm in width and 0.07–0.40 mm in thickness, while their length remains indefinitely continuous, attributed to the inherent elasticity and amorphous nature of the material^39^. The XRD pattern of the alloys exhibits broad and low-intensity diffraction peaks, characteristic of an amorphous phase, within the 2θ range of 40–50°. Notably, a prominent diffraction peak is observed at 2θ ≈ 45°, which corresponds to the signature peak of Fe-based alloys^40^indicating the presence of crystalline Fe-based phases amidst the predominantly amorphous matrix.

Differential Scanning Calorimetry (DSC) analysis was performed to evaluate the glass-forming ability (GFA) and thermodynamic properties of the samples. The resulting DSC curves are presented in Fig. 1(b)-(c) obtained at a heating rate of 20 K/min from room temperature to 1750 K for Hf4 and Hf6 alloys. GFA of Hf4 and Hf6 alloys are carried out using a theoretical approach by Rao et al. and a new thermodynamic parameter $\:{P}_{HSS}$ parameter which consolidates a comprehensive set of thermodynamic factors that significantly impact GFA into a singular metric which is given in the Eq. (1).

1 $$\:{P}_{HSS}={\varDelta\:H}^{C}\left(\frac{{\varDelta\:S}_{C}}{R}\right)\left(\frac{{\varDelta\:S}_{\sigma\:}}{{k}_{B}}\right)$$.

Where the enthalpy of chemical mixing ($\:{\varDelta\:H}^{C}$) quantifies the energetic interactions between constituent atoms, while configurational entropy ($\:{\varDelta\:S}_{C}$) is exclusively determined by the atomic fractions of the constituent elements, independent of alloy-specific factors. Conversely, mismatch entropy ($\:{\varDelta\:S}_{\sigma\:}$) emerges as a critical factor, driven by atomic size disparities that inherently influence the alloy’s thermodynamic stability. The incorporation of fundamental physical constants, namely the gas constant ($\:R$) and Boltzmann constant ($\:{k}_{B}$), ensures a comprehensive and physically informed framework for predicting the alloy’s thermodynamic properties^41^.$\:{P}_{HSS}$ offers the distinct advantage of predicting GFA without the necessity of synthesizing the alloy.

The incorporation of Hf into Hf4 and Hf6 alloys results in a decrease in GFA, which suggests a decrease in amorphous nature. The thermodynamic parameters support this trend. It is important to note that the packing efficacy of Hf is disrupted by its slightly larger atomic radius (157.5 pm) in comparison to that of Zr (155.8 pm), which favors crystallinity^42^. Moreover, the thermodynamic drive for amorphization is reduced by the fact that the enthalpy of mixing (ΔH_mix_) between Hf and other alloy elements is less negative than that of Zr^43^ Table 1.

**Table 1:** Summary of various thermodynamic parameters.

Material	$\:{\varDelta\:\varvec{H}}_{\varvec{C}}$	$\:\frac{\varDelta\:{\varvec{S}}_{\varvec{C}}}{\varvec{R}}$	$\:\frac{\varDelta\:{\varvec{S}}_{\varvec{\sigma\:}}}{{\varvec{k}}_{\varvec{B}}}$	$\:{\varvec{P}}_{\varvec{H}\varvec{S}\varvec{S}}$
Hf4	−11.16	0.521	0.045	−0.264
Hf6	−10.91	0.502	0.015	−0.084

### Magnetic properties

Temperature-dependent magnetization curves for Hf4 and Hf6 alloys under 100 Oe magnetic field were obtained to elucidate their magnetic behavior and phase transformation, as shown in Fig. 1 (d)-(e). As evident from the insets of Fig. 1 (d)-(e), the magnetization of both alloys still exhibits a rapid decrease, eventually approaching zero as the temperature increases, indicating their monophase amorphous alloy nature at measured temperatures was determined by analyzing the $\:\frac{dM}{dT}\:$plots obtained from the M-T curves of $\:{T}_{C}\:$values^32^ ​​of 293 K and 303 K were reported for the alloys Hf4 and Hf6, respectively, as shown in the inset of Fig. 1 (d)-(e). The substitution of Hf for Zr in the Hf4 and Hf6 amorphous alloys yields a significant increase in the Curie transition temperature ($\:{T}_{C}$). This enhancement is due to subtle changes in electronic structure and magnetic properties resulting from differences in atomic size and density between Hf and Zr, despite their similar atomic radii.

In addition, a linear fitting according to the Curie-Weiss law^44^ was performed using the reciprocal of magnetic susceptibility (χ − 1) data of the paramagnetic (PM) field, and the resulting fitting curves are shown in the inset Fig. 1 (d)-(e). The Curie-Weiss temperatures, θ_CW_, for the Fe-Zr-Hf-B-Cu alloys, were calculated to be 293 K and 303 K. The $\:{T}_{C}$values ​​obtained from this method are the same as the $\:{T}_{C}$values ​​determined from the M-T curve match well, indicating the reliability and robustness of the magnetic phase transition temperature.

### Magnetocaloric effect

**Fig. 2:**
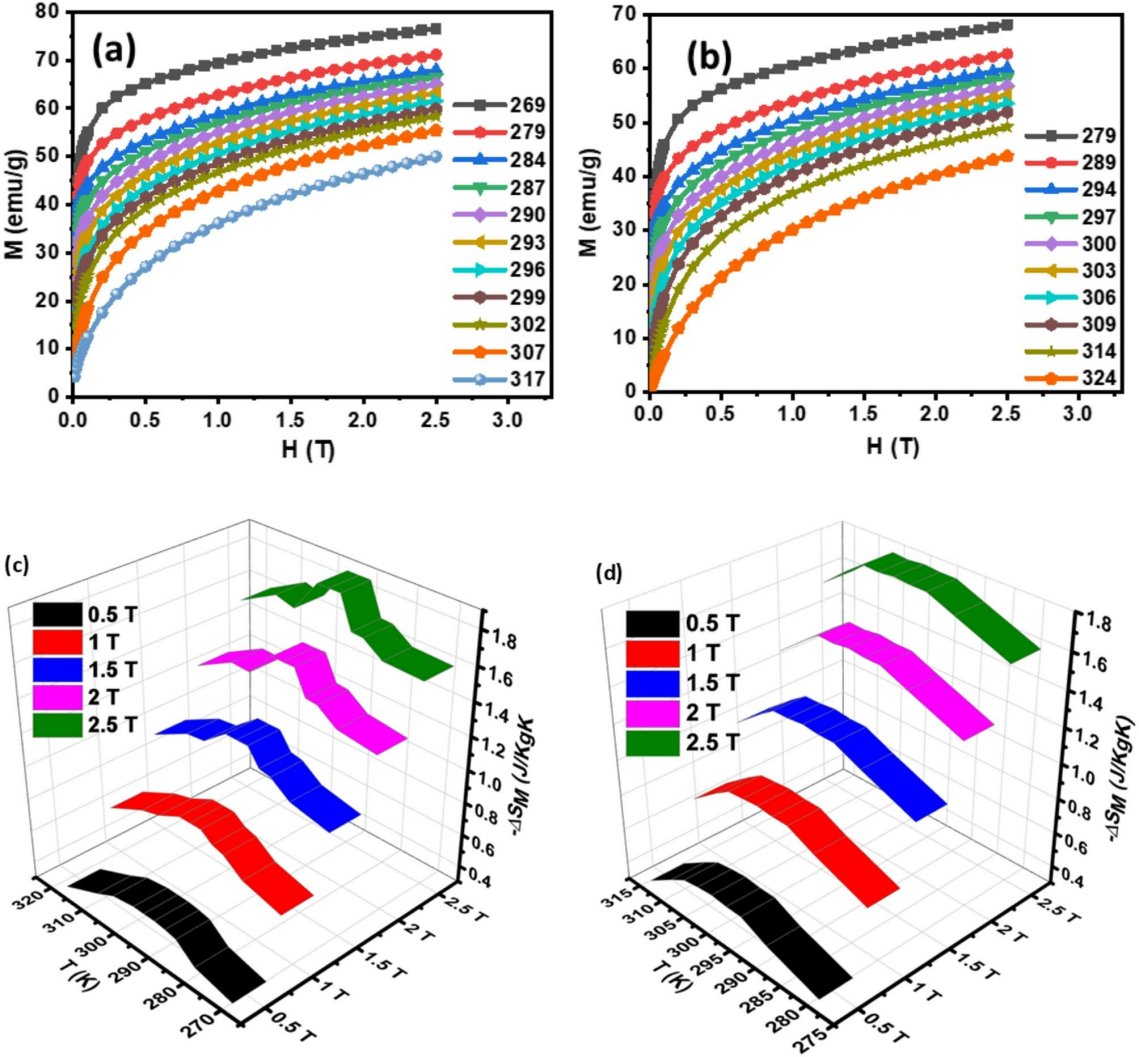
**(a)-(b)** M-H data around Curie transition temperature of Hf4 and Hf6 ribbons **(c)-(d)** magnetic entropy versus temperature curves of Hf4 and Hf6 ribbons.

To elucidate the magnetic properties and phase transitions of Hf4 and Hf6 alloys near its Curie transition temperature ($\:{T}_{C}$), a comprehensive investigation of isothermal magnetization curves (M-H curves) was conducted over a broad temperature range of 269–317 K for Hf4 alloys and 279–324 K for Hf6 alloys. The resulting M-H curves are presented in Fig. 2 (a)-(b), providing valuable insights into the temperature-dependent magnetic properties of the material. The measured M-H curves reveal an increasing order in magnetization with decreasing temperature in the studied range. This phenomenon can be attributed to the low-temperature fluctuation of the spin at low temperatures, which increases the magnetic order of the material. Notably, the temperature increment was optimized to 3 K within the critical range of 284–302 K for Hf4 alloys and 294–309 K for Hf6 alloys, while larger increments (5–10 K) were employed for other temperature ranges, ensuring a balance between data resolution and experimental efficiency. The observed temperature-dependent magnetization behavior is consistent with expectations for ferromagnetic materials, where reduced thermal agitation enables stronger magnetic correlations^45^.

Magnetocaloric materials exhibit an intrinsic property called the magnetocaloric effect (MCE), which is quantified by the magnetic entropy change ($\:{\varDelta\:S}_{M}$) close to the Curie transition temperature ($\:{T}_{C}$). This temperature-dependent phenomenon has the expression essential for advanced cooling technology. In order to evaluate the MCE properties, need to calculate $\:{\varDelta\:S}_{M}$ from the isothermal magnetization curves (M-H curves) using established thermodynamic relationships.

The Magnetocaloric Effect (MCE) is an inherent characteristic of magnetic materials, quantifiable through the $\:{\varDelta\:S}_{M}$ in the vicinity of the Curie transition temperature ($\:{T}_{C}$). The Maxwell relation offers a robust framework for quantitatively evaluating the magnitude of $\:{\varDelta\:S}_{M}$that arises from the application of a magnetic field (H). Mathematically, the Maxwell relation is expressed in the Eq. (8)^46^

8 $$\:{\varDelta\:S}_{M}=\:{\int\:}_{0}^{H}\frac{dM}{dT}dH$$.

The calculated $\:{\varDelta\:S}_{M}$values ​​for all models consistently showed negative trends in the temperature and magnetic fields investigated. These models show a reversible magnetization process. Negative $\:{\varDelta\:S}_{M}$ values ​​indicate that magnetic entropy increases with decreasing temperature. The magnitude of -$\:{\varDelta\:S}_{M}\:$reveals a strong dependence on temperature and magnetic field.

Figure 2 (c)-(d) presents the temperature-dependent variation of the -$\:{\varDelta\:S}_{M}\:$at various magnetic fields up to 2.5 T. Table 2 summarizes the peak values of the $\:{\varDelta\:S}_{M}\:$curve, denoted as $\:{\varDelta\:S}_{M}^{peak}$, and the corresponding peak temperature (T^peak^)^47^ at a magnetic field of 2.5 T. A systematic $\:{\varDelta\:S}_{M}^{peak}$increase in is observed with increasing Hf atomic percentage. This correlation can be attributed to the enhanced magnetization at the maximum magnetic field, resulting from the increased Hf concentration. The larger magnetization contributes to a more pronounced magnetic entropy change, thereby elevating $\:{\varDelta\:S}_{M}^{peak}$. The $\:{\varDelta\:S}_{M}^{peak}$ values, representing the peak magnetic entropy change, are found to coincide with or occur proximal to the Curie transition temperature ($\:{T}_{C}$). This observation underscores the significance of $\:{T}_{C}$ in determining the magnetocaloric properties.

The material’s suitability for magnetic refrigeration technology was assessed by evaluating the full-width at half-maximum ($\:{{\Delta\:}T}_{FWHM}$) of the peak magnetic entropy change. This metric represents the working temperature span of the material under a given magnetic field. Notably, at 2.5 T, $\:{{\Delta\:}T}_{FWHM}$ was calculated to be 12.59 K and 31.98 K for Hf4 and Hf6 alloys respectively. This value signifies the temperature range over which the material exhibits a substantial magnetic entropy change, making it a promising candidate for magnetic refrigeration applications. A larger $\:{{\Delta\:}T}_{FWHM}$ indicates a broader operating temperature range, enhancing the material’s potential for efficient cooling. The observed $\:{{\Delta\:}T}_{FWHM}$ value suggests that the material can effectively facilitate heat transfer and magnetic entropy change within a temperature span of 12.59 K for Hf4 alloys and 31.98 K for Hf6 alloys. This characteristic is crucial for optimizing magnetic refrigeration systems.

A vital statistic for evaluating the magnetocaloric performance of materials is the Relative Cooling Power (RCP), a measure that quantifies the material’s heat transfer capability. The RCP is determined by multiplying the peak magnetic entropy change ($\:{\varDelta\:S}_{M}^{peak}$) by the full-width at half-maximum ($\:{{\Delta\:}T}_{FWHM}$) of the peak^48^.

9 $$\:RCP=\left|-{\varDelta\:S}_{M}^{peak}\right|*{{\Delta\:}T}_{FWHM}$$.

**Table 2:** Different magnetocaloric parameters.

Material	$\:{\varvec{T}}_{\varvec{C}}\:\left(\varvec{K}\right)$	$\:{-\varDelta\:\varvec{S}}_{\varvec{M}}^{\varvec{m}\varvec{a}\varvec{x}}\left(\frac{\varvec{J}}{\varvec{k}\varvec{g}\varvec{K}}\right)$	$\:\varvec{R}\varvec{C}\varvec{P}\:\left(\frac{\varvec{J}}{\varvec{k}\varvec{g}}\right)$	$\:{\varvec{\Delta\:}\varvec{T}}_{\varvec{F}\varvec{W}\varvec{H}\varvec{M}\:}\left(\varvec{K}\right)$	T^peak^ (K)
Hf4	293	1.856	23.36	12.59	293
Hf6	303	1.767	56.50	31.98	303

These parameters provide a comprehensive assessment of the magnetocaloric potential of the material, taking into account the magnitude of the magnetic entropy change and the operating temperature. The higher RCP value indicates a higher cooling rate of the Hf6 alloy.

## Critical scaling

**Fig. 3:**
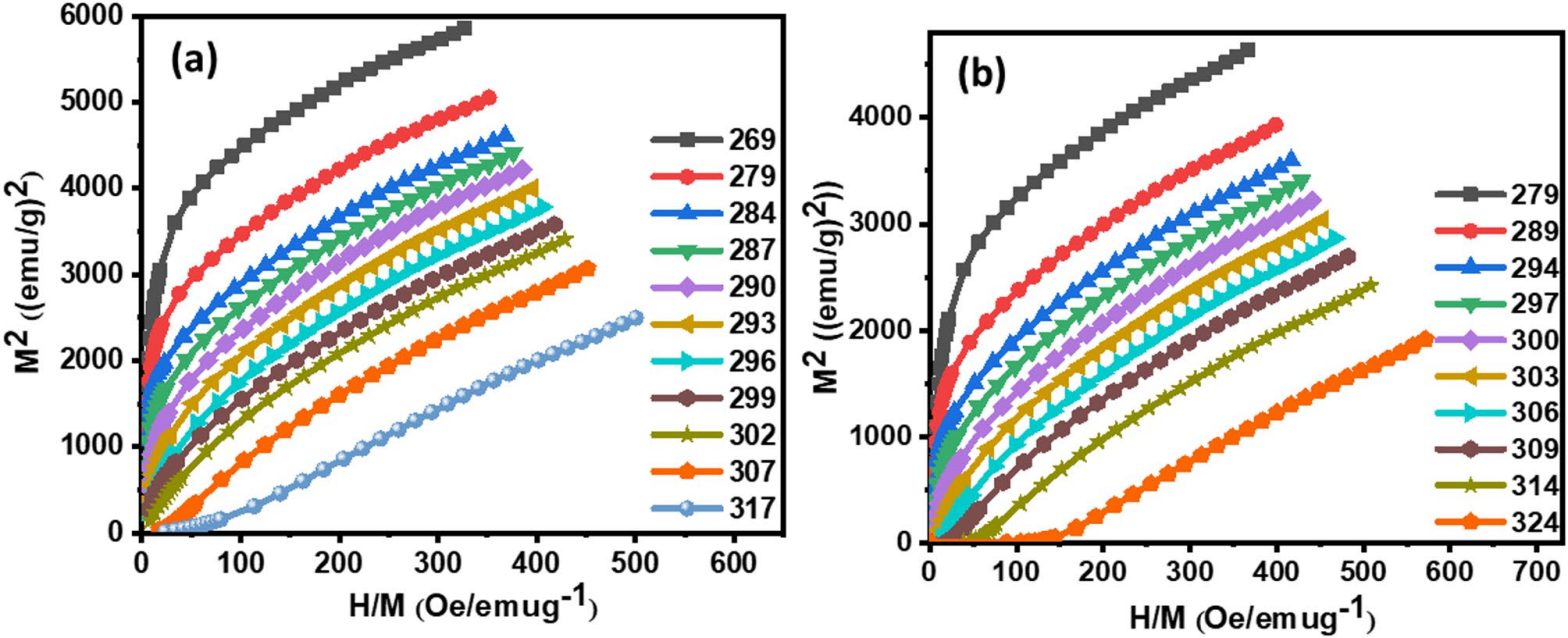
**(a)** Arrott curves of Hf4 ribbons **(b)** Arrott curves of Hf6 ribbons.

The magnetocaloric properties of Hf4 and Hf6 alloys compounds are intricately linked to their magnetic phase transition characteristics. To elucidate the transition types, Arrott plots ($\:{M}^{2}$ versus $\:\frac{H}{M}$) were constructed from isothermal magnetization curves, as depicted in Fig. 3 (a)-(b). The Banerjee criterion provides a framework for interpreting these plots, where negative slopes signify first-order magnetic phase transitions (FOMPT), and positive slopes indicate second-order transitions (SOMPT)^49^.

The Arrott plots exhibit positive slopes below the respective Curie transition temperatures, suggestive of SOMPT characteristics. This positivity at $\:{T}_{C}$ is crucial, as it corresponds to the primary working temperature range for magnetocaloric materials. Consequently, Hf4 and Hf6 alloys are classified as SOMPT materials, ensuring favorable thermal and magnetic reversibility. This reversibility is essential for optimizing magnetocaloric performance.

The scaling hypothesis provides a framework for understanding the SOMPT occurring near the Curie transition temperature ($\:{T}_{C}$). This transition is characterized by a set of critical exponents that govern the universal behavior of the system at the critical point. Specifically, the spontaneous magnetization exponent ($\:\beta\:$), susceptibility exponent ($\:\gamma\:$), and magnetic field exponent ($\:\delta\:$) define the SOMPT^50^.

This transformation is based on the magnetic equation of state, which delineates the correlation among magnetization, magnetic field, and temperature in proximity to the critical point. Through the examination of these graphs, we may ascertain the critical exponents β and γ, which define spontaneous magnetization and susceptibility, respectively.

The critical exponents are quantitatively expressed in Eqs. (10)–(12), which delineate the relationships among magnetization, susceptibility, and magnetic field in proximity to $\:{T}_{C}$. The parameters β, γ, and δ govern the characteristics of the phase transition, influencing the system’s behavior around the critical point. Through the analysis of these exponents, researchers can acquire insights into the fundamental physics that drives the SOMPT, hence guiding the creation of materials with customized magnetic characteristics for diverse purposes^51,52^.

10 $$\:{M}_{sp}\left(T\right)={M}_{0}{\left(-\epsilon\:\right)}^{\beta\:},for\:\epsilon\:<0$$

11 $$\:{\chi\:}_{0}^{-1}\left(T\right)=\left(\frac{{h}_{0}}{{m}_{0}}\right){\left(\epsilon\:\right)}^{\gamma\:},for\:\epsilon\:>0$$

12 $$\:M\left({T}_{C}\right)=D{H}^{\frac{1}{\delta\:}}\:,\:for\:\:\epsilon\:=0\:\:\:\:\:\:\:\:\:\:\:\:\:\:\:$$.

where $\:\epsilon\:\:=\:\frac{(T\:-\:{T}_{c})\:}{{T}_{c}}$ indicates the reduced temperature, $\:{\chi\:}_{0}^{-1}\:$is the inverse zero-field susceptibility, D is the critical amplitude, H is the applied magnetic field, and $\:{M}_{sp}$, $\:{m}_{0}$, $\:{h}_{0}$ and $\:{m}_{0}$ indicates the spontaneous magnetization, and critical amplitude respectively.

A comprehensive analytical framework was developed to investigate the critical behavior of the alloys, leveraging the strengths of multiple techniques. By combining Modified Arrott Plots (MAP), Kouvel-Fisher (KF) analysis, Critical Isotherm Analysis (CIA), and Widom Scaling Relation (WSR)^53^, a detailed picture of the magnetic phase transition dynamics emerged. The synergistic approach enabled the precise determination of critical exponents ($\:\beta\:$, $\:\gamma\:$, $\:\delta\:$) and the Curie transition temperature ($\:{T}_{C}$), providing valuable insights into the alloys’ magnetic behavior. The combined analysis revealed the intricate relationships between spontaneous magnetization, susceptibility, and magnetic field, ultimately enhancing our understanding of the complex phenomena governing magnetic phase transitions in Hf4 and Hf6 alloys.

To initiate our investigation, we utilized Arrott-Noakes plots, also known as Modified Arrott Plots (MAPs), to estimate the critical exponents ($\:\beta\:$, $\:\gamma\:$) and Curie transition temperature ($\:{T}_{C}$). This analytical technique involves transforming magnetization (M) versus magnetic field (H) data into isothermal plots of $\:{\left(\frac{H}{M}\right)}^{\frac{1}{\gamma\:}}$ versus $\:{M}^{\frac{1}{\beta\:}}$ which is shown in the Eq. (13).

13 $$\:{\left(\frac{H}{M}\right)}^{\frac{1}{\gamma\:}}=a\left(\frac{T-{T}_{c}}{T}\right)+b{\left(M\right)}^{\frac{1}{\beta\:}}$$.

Where a and b are constants.

**Fig. 4:**
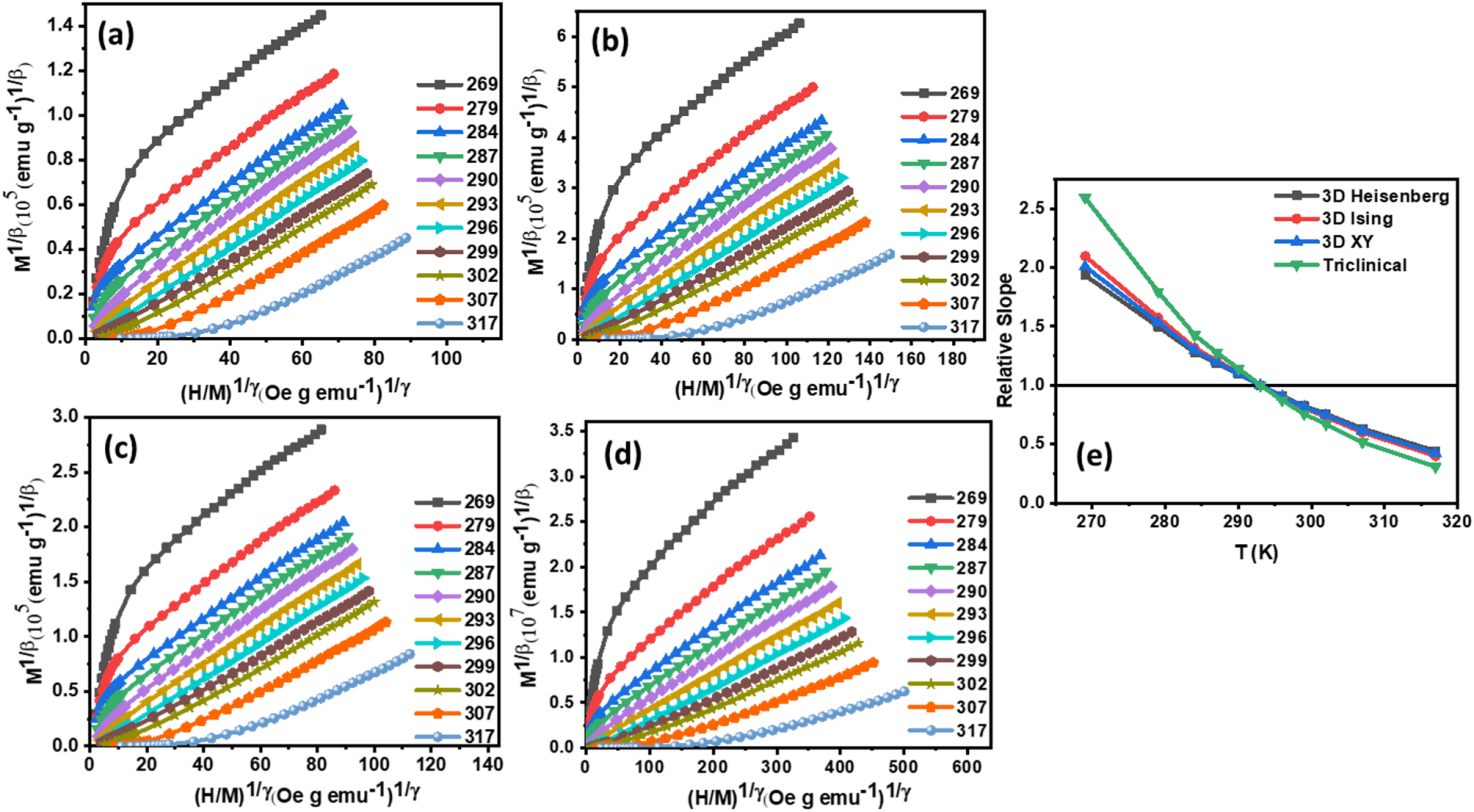
**(a)** 3D Heisenberg Model curves **(b)** 3D Ising Model curves **(c)** 3 XY Model curves **(d)** Tricritical Model curves **(e)** Relative slope of all models of Hf4 ribbons.

**Fig. 5:**
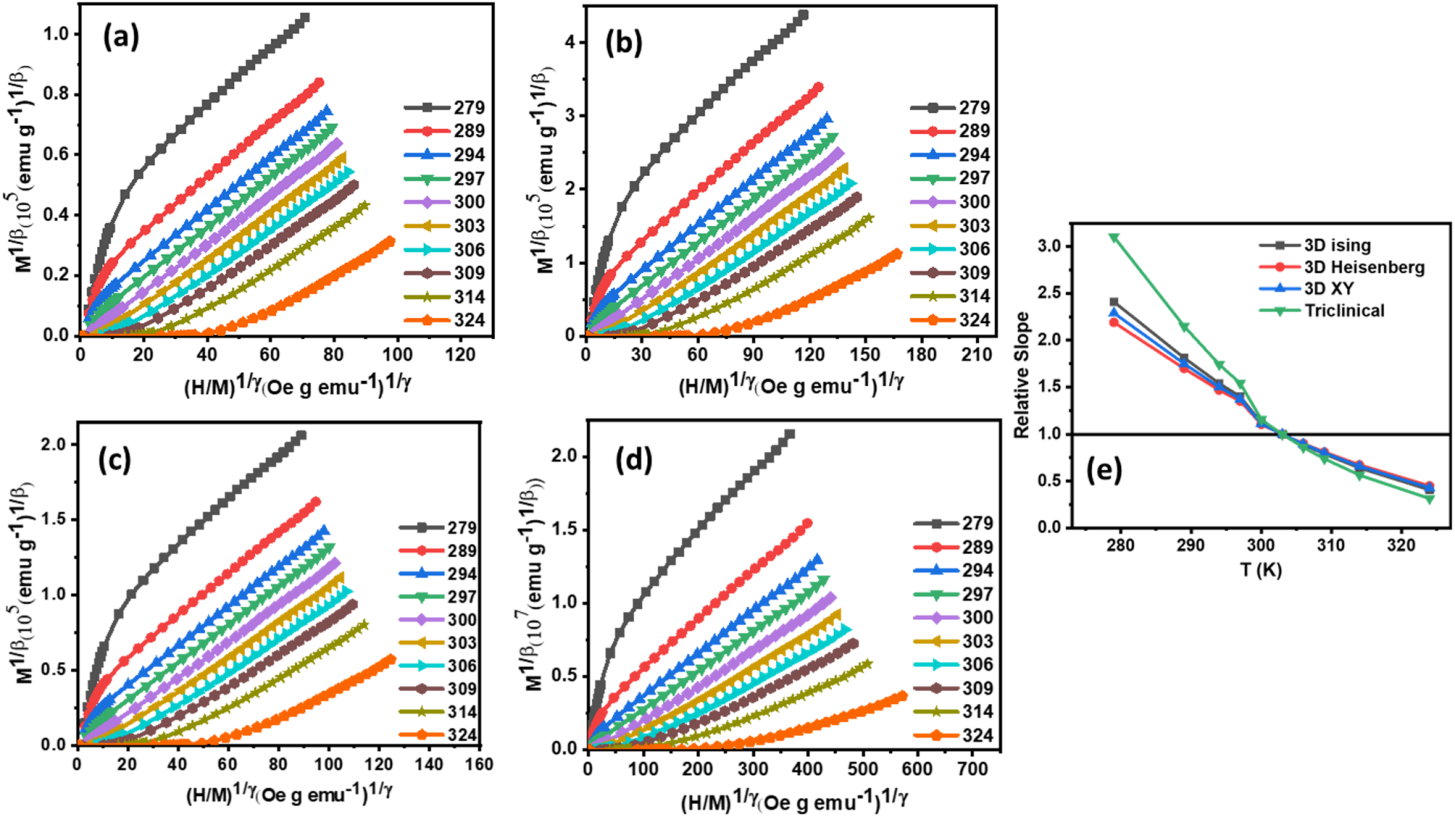
**(a)** 3D Heisenberg Model curves **(b)** 3D Ising Model curves **(c)** 3D XY Model curves **(d)** Tricritical Model curves **(e)** Relative slope of all models of Hf6 ribbons.

The magnetic equation of state is graphically represented in Fig. 4 (a)-(d) and Fig. 5 (a)-(d), showcasing $\:{\left(\frac{H}{M}\right)}^{\frac{1}{\gamma\:}}$ versus $\:{M}^{\frac{1}{\beta\:}}$ plots at multiple temperatures for four theoretical frameworks:3D-Heisenberg model with $\:\beta\:$ = 0.365 and $\:\gamma\:$ = 1.386 values, 3D-Ising model with $\:\beta\:$ = 0.325 and $\:\gamma\:$ = 1.24 values, 3-XY models with $\:\beta\:$ = 0.345 and $\:\gamma\:$ = 1.316 values and Tricritical mean-field model with $\:\beta\:$ = 0.25 and $\:\gamma\:$ = 1 values. This comparative analysis facilitates the assessment of each model’s efficacy in describing the magnetic phase transition dynamics^51^.

To quantitatively evaluate the efficacy of each theoretical model in describing the magnetic behaviour of the compounds, we calculated the relative slopes (RS) of the Arrott-Noakes plots in the high-field region. This analysis, presented in Fig. 4 (e) and Fig. 5 (e) enabled a systematic comparison of the models and identification of the optimal descriptor for both compounds. The relative slopes were computed using the expression in the Eq. (14).

14 $$\:RS=\frac{S\left(T\right)}{S\left({T}_{C}\right)}$$.

This quantitative assessment facilitated the discernment of subtle differences between the models, ultimately informing the selection of the most suitable theoretical framework for capturing the complex magnetic phase transition dynamics exhibited by the compounds. where $\:S\left(T\right)$ and $\:S\left({T}_{C}\right)$ are the isotherm slopes around and at the Curie transition temperature ($\:{T}_{C}$) for each theoretical model^53^.

The suitability of each theoretical model was assessed by examining the relative slope (RS) values, with values approaching unity (RS ≈ 1) indicating optimal agreement with experimental data. As shown, the 3D-Heisenberg model consistently exhibits RS values nearest to unity, outperforming alternative models in terms of accuracy and reliability. This outcome underscores the 3D-Heisenberg model’s efficacy in describing the complex magnetic behavior of the system.

**Fig. 6:**
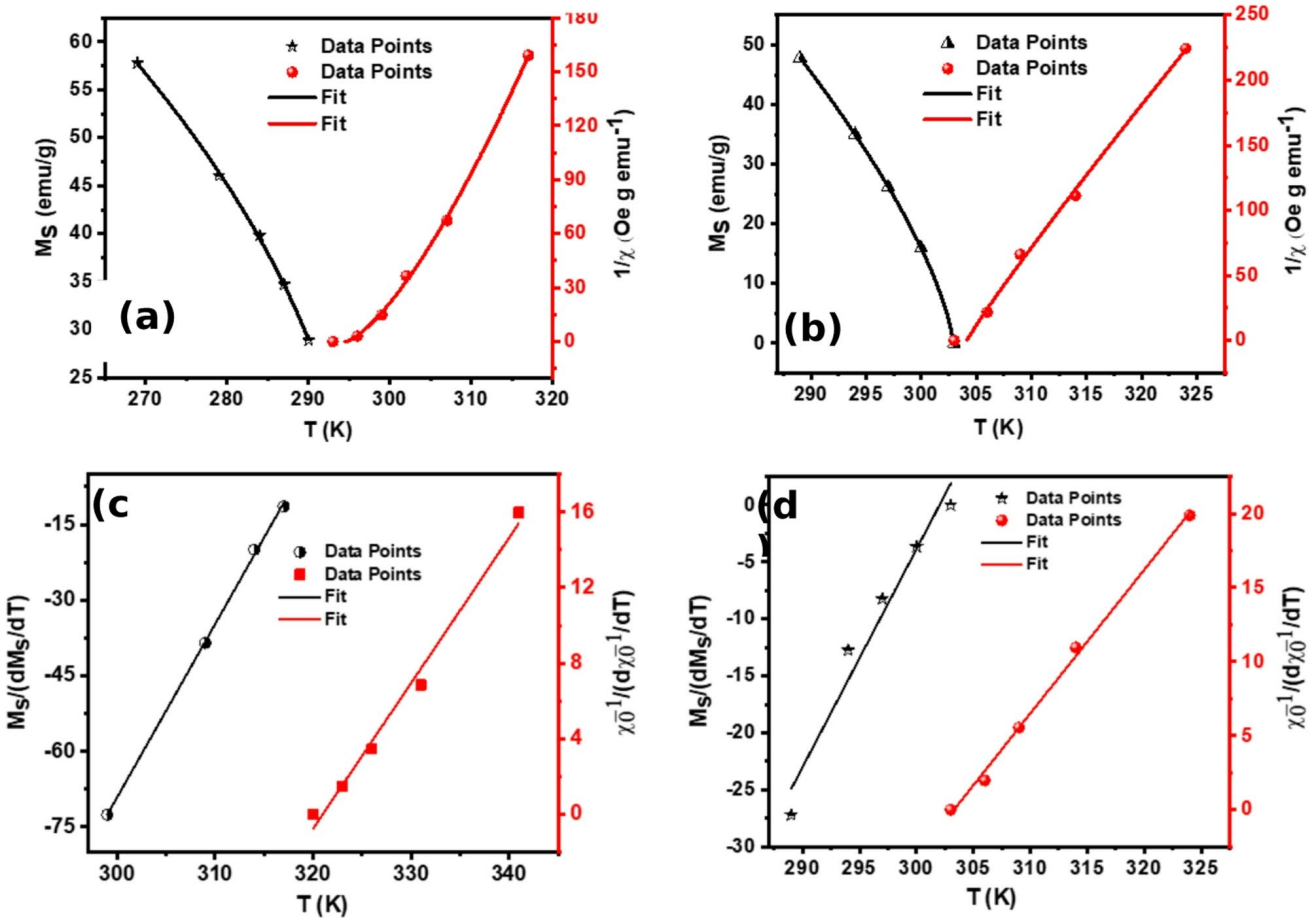
Modified Arrott Plots of **(a)** Hf4 ribbons and **(b)** Hf6 ribbons. Kouvel–Fisher plots of **(c)** Hf4 ribbons and **(d)** Hf6 ribbons.

Utilizing the MAP technique, spontaneous magnetization values, $\:{M}_{sp}\left(T\right)$, and inverse initial susceptibility, $\:{\chi\:}_{0}^{-1}\left(T\right)$, were determined through linear extrapolation to intersect with the $\:{\left(\frac{H}{M}\right)}^{\frac{1}{\gamma\:}}$ and $\:{M}^{\frac{1}{\beta\:}}$ axes, respectively. The resultant values are compiled in Table 3. Concurrently, critical exponents were extracted by fitting Eqs. (10)-(11) via the MAP method, with the corresponding plots presented in Fig. 6(a)-(b). This comprehensive analysis enables a precise evaluation of the magnetic phase transition dynamics.

A complementary analysis was performed using the Kouvel-Fisher (KF) approach to reassess the critical exponents $\:\beta\:$, $\:\gamma\:$, and $\:\delta\:$, based on Eqs. (15)–(16). The corresponding KF plots are displayed in Fig. 6(c)-(d), offering a cross-validation of the extracted critical exponent values^54^.

15 $$\:\left[\frac{{M}_{s}}{\frac{d{M}_{s}}{dT}}\right]=\frac{[T-{T}_{c}]}{\beta\:}$$

16 $$\:\left[\frac{{\chi\:}_{0}^{-1}}{\frac{d{\chi\:}_{0}^{-1}}{dT}}\right]=\frac{[T-{T}_{c}]}{\gamma\:}$$.

where $\:{T}_{C}$ is the critical temperature, $\:\beta\:$ and $\:\gamma\:$ are the critical exponents, $\:{\chi\:}_{0}^{-1}\left(T\right)$ is the inverse initial susceptibility, and $\:{M}_{S}$(T) is the spontaneous magnetization, as indicated in Table 3.

**Fig. 7:**
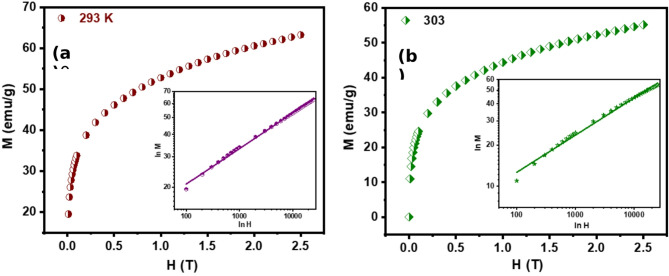
MH plot **(a)** at 293 K of Hf4 ribbons and **(b)** 303 K of Hf6 ribbons. Inset curves depict the same plot in ln-ln scale.

To determine the critical exponent $\:\delta\:$, we analyzed the critical isotherm at T = $\:{T}_{C}$ using a logarithmic plot of M versus H. The resulting linear relationship, characterized by a slope of $\:\frac{1}{\delta\:}$, validated the predicted power-law dependence. This finding is illustrated in Fig. 7 (a)-(b).

The critical exponents $\:\beta\:$, $\:\delta\:$, and $\:\gamma\:$ adhere to the Widom scaling relation^55^which dictates that these exponents are interrelated through the Eq. (17).

17 $$\:\delta\:=1+\:\frac{\gamma\:}{\beta\:}$$.

The remarkable agreement between $\:\delta\:$ values obtained from the Widom scaling relation and those extracted from critical isotherms at $\:{T}_{C}$provides strong evidence for the self-consistency and precision of our estimated $\:\beta\:$ and $\:\gamma\:$ critical exponents. This concordance demonstrates the robustness of our analytical approach.

**Table 3:** Analysis of various theoretical frameworks.

Sample/model	Method	β	γ	δ	Ref.
Hf4	MAP	0.483	1.378		Present work
	KF	0.471	1.297		Present work
	CIA			4.95	Present work
Hf6	MAP	0.698	0.932		Present work
	KF	0.522	1.031		Present work
	CIA			3.67	Present work
3D Heisenberg		0.365	1.336	4.80	^51^
3D Ising		0.325	1.24	4.82	^51^
3 XY		0.345	1.316	4.81	^51^
Mean Field		0.5	1	3	^51^
Tricritical		0.25	1	5	^51^

## **Conclusion**

In summary, our comprehensive investigation of Fe-Zr-Hf-B-Cu alloys has provided profound insights into their structural, thermal, magnetic, and magnetocaloric properties. X-ray diffraction measurements unequivocally confirmed the amorphous nature of the alloy. Notably, Arrott plot analysis, interpreted through the Banerjee criterion, revealed a second-order magnetic phase transition at the critical temperature ($\:{T}_{C}$), characterized by a positive slope. Employing a multi-faceted analytical approach, combining Arrott plots (MAP), Kouvel-Fisher (KF) analysis, critical isotherm analysis (CIA), and Widom scaling relation (WSR) methods, we accurately determined the critical exponents $\:\beta\:$, $\:\gamma\:$, and $\:\delta\:$. The obtained exponents conform to the mean-field model and satisfy scaling relations, ensuring the reliability and authenticity of our results. Most significantly, our magnetocaloric measurements reveal magnetic entropy change of 1.856 for Fe_88_Zr_3_Hf_4_B_4_Cu_1_ alloy and 1.767 J/kgK for Fe_88_Zr_1_Hf_6_B_4_Cu_1_ alloy in response to a 2.5 T field change at 293 K room temperature (303 K) respectively. Furthermore, the full-width at half-maximum ($\:{{\Delta\:}T}_{FWHM}$) of 12.59 K and 31.98 K and the Refrigerant Capacity ($\:RCP$) of the Fe_88_Zr_3_Hf_4_B_4_Cu_1_ alloy had 23.36 J/kg at 293 K and Fe_88_Zr_3_Hf_2_B_4_Cu_1_ alloy reached a remarkable value of 56.50 J/kg at 303 K, underscoring its potential as a promising candidate for magnetic refrigeration applications at room temperature.

## Data Availability

The corresponding author can provide the datasets used and/or analyzed in the current work upon reasonable request.
